# An Exchange with "The Hospital."

**Published:** 1921-08-20

**Authors:** 


### An Exchange with "The Hospital."

*To the Editor of* The Hospital.

Sir,—In answer to your request from a nurse to exchange a copy of *The Nursing Mirror* every week for The Hospital, I should be pleased to post the latter weekly on Tuesdays to her in exchange.—Yours truly,

Ex "Q.A.I.M.N.S."

[We have put these correspondents in communication with each other, and hope the exchange will bring them both much interest and satisfaction.]

